# The Irish cattle population structured by enterprise type: overview, trade & trends

**DOI:** 10.1186/s13620-022-00212-x

**Published:** 2022-04-04

**Authors:** Jonas Brock, Martin Lange, Jamie A. Tratalos, Natascha Meunier, Maria Guelbenzu-Gonzalo, Simon J. More, Hans-Hermann Thulke, David A. Graham

**Affiliations:** 1 Helmholtz Centre for Environmental Research GmbH - UFZ, PG Ecological Epidemiology, Leipzig, Germany; 2 Animal Health Ireland, Carrick-on-Shannon, Co. Leitrim Ireland; 3 Centre for Veterinary Epidemiology and Risk Analysis, UCD School of Veterinary Medicine, University College Dublin, Dublin, D04 W6F6 Ireland

**Keywords:** Livestock, Cattle, Herd type, Ireland, Herd classification, Dairy, Beef, Production

## Abstract

**Background:**

The cattle sector is the most important economic production unit of the Irish farming and agri-food sector. Despite its relevance, there has been limited quantitative information about the structure of differing cattle production types and of the connections between them. This paper addresses this gap by providing, for the first time, an overview of the Irish cattle population structured by enterprise type.

**Methods & Results:**

We collected data from the cattle register for the period 2015 to 2019 and assigned registered herds to one of 18 different herd types using a recently published herd type classification approach. This allows, for the first time, to exploring changes in enterprise types and subtypes over time, and describing the movements between these subtypes and from these subtypes to slaughter.

**Conclusions:**

The overview and associated classification presented in this study will form the basis for a number of future comparative studies, including cross-sectoral assessments of profitability, estimation of the extent of animal health losses on Irish cattle farms or structural analysis of Greenhouse Gas (GHG) emissions across production systems.

**Supplementary Information:**

The online version contains supplementary material available at 10.1186/s13620-022-00212-x.

## Introduction

The cattle sector in Ireland, from farm to processing and export is an important indigenous industry and a vital part of the agri-food sector. In 2019, there were approximately six and a half million cattle in Ireland, kept on more than 100.000 farms. In the same year, total annual Irish milk production was around 7.9 billion litres [1], with the majority of milk and associated products being exported, to the value of €4.4 billion annually [2]. Further, Ireland is one of the largest exporters of beef, along with Australia, Brazil and USA. In 2019, more than 624.000 t of beef were produced, leading to beef exports worth approximately €2.1 billion [3].

Despite the importance of these industries to the national economy, there has been limited quantitative information about the different cattle production types within Ireland, and of the connections between them. This information is useful to inform discussion on a range of relevant issues, including a better understanding of disease epidemiology in infectious disease eradication planning and surveillance.

A qualitative understanding of differing enterprise types within the major production systems of beef and dairy is recognised. For example, some dairy farms raise most of their replacement animals and keep surplus animals for fattening, while others introduce new heifers or cows from outside the herd and sell animals not used for milk production [4]. Further, the process of contract rearing of e.g. dairy heifers, where they are raised in external herds and then returned to their birth herd prior to first calving, has become increasingly common in recent years [5, 6]. Likewise, a variety of beef production systems are recognised [4, 7]. To this point, however, quantitative information about the structure and connections of differing cattle enterprise types has been limited to population numbers and animal movements in the Irish cattle sector [8, 9].

In Ireland, demographic cattle data at the animal and herd level is available through the Animal Identification and Movement database (AIM) maintained by the Department of Agriculture, Food and the Marine (DAFM), but is primarily categorised at the level of beef and dairy [10]. The Irish Cattle Breeding Federation (ICBF; www.icbf.com) classifies breeding herds into dairy, beef and mixed. Due to the specialization of Irish cattle farming this classification rarely provides the level of detail required for a number of applications, including epidemiological risk analysis and effective policy development. This is particularly evident in the area of animal health management, most recently through the introduction of the European Animal Health Law (AHL). For the bovine sector, with the new EU regulations the herd management type became an important criterion for shaping the sampling strategy of national surveillance and control programs. With regard to BoHV-1 eradication as an example, the AHL prescribes certain rules on how herds can achieve disease-free status. These rules often interact with the specifics of herd management going beyond the categories of dairy, beef and mixed herds.

Brock et al [11] recently published a study introducing a new approach to herd classification that captures the specialisation of the Irish cattle sector. This approach combines formal similarity analysis (machine-learning methods) with non-formal knowledge-based interpretation. It exploits multi-dimensional herd registry data, visualises similar farming principles and iterates on visually identified inconsistencies until a comprehensive classification scheme is achieved. By this approach, classification rules were derived in consultation with Irish livestock experts to assign each herd in Ireland to one of 17 different enterprise types. Here, we apply this classification to the AIM database between 2015 and 2019, with the objectives of generating an overview of the Irish cattle population, exploring changes in enterprise types and subtypes over this period, and describing the movements between these subtypes and from these subtypes to slaughter.

## Methods

### Datasets & data handling

Data for this analysis were obtained from the AIM database maintained by DAFM. In the AIM database, each registered animal in the Republic of Ireland is listed, along with its unique tag number (ID) so that its sex, breed, and birth date, as well as the identity of its birth herd and dam, can be ascertained.

Calving in most Irish herds is seasonal, with a strong bias to spring calving with peak numbers born in February [12]. Therefore, we accessed the database for three dates (1st Jan, 1st May & 1st September) in 2015, 2016, 2017, 2018 and 2019, respectively, and extracted the demographic data for all cattle listed on these dates. This approach was chosen to get an impression of the herd composition at different times of the year instead of a single snapshot.

The AIM data is available at the animal level, meaning each animal is recorded with its own unique identity (ID) or tag number, along with its sex, breed and birth date, as well as the IDs of its herd and dam. These data were aggregated at the herd level so that we had one dataset for each year comprising those herds with one or more registered animals present during at least one of the time points of interest. While aggregating the data at the herd level a number of demographic decision parameters were calculated for each individual herd (in summary, the proportions of animals that are: female; of dairy breed; of dairy-beef cross breed; have ever produced a calf; male between 1 and 2 years of age; purchased animals that remain for less than 30 days (relative to all out moves each year). The variables were chosen according to the classification scheme proposed by [11]. In a next step movement data was extracted for all days from 1st January 2015 to 31st December 2019. Again, transport-related decision parameters (in summary, the proportions of moves out of the herd which went to slaughter or to the herd of birth of the departing animals, and the proportion of moves in represented animals returning to their birth herd) were calculated for each year and herd according to [11]. A detailed description of the calculated parameters can be found in [11] and Additional file 1.

### Herd type classification

**Fig. 1:**
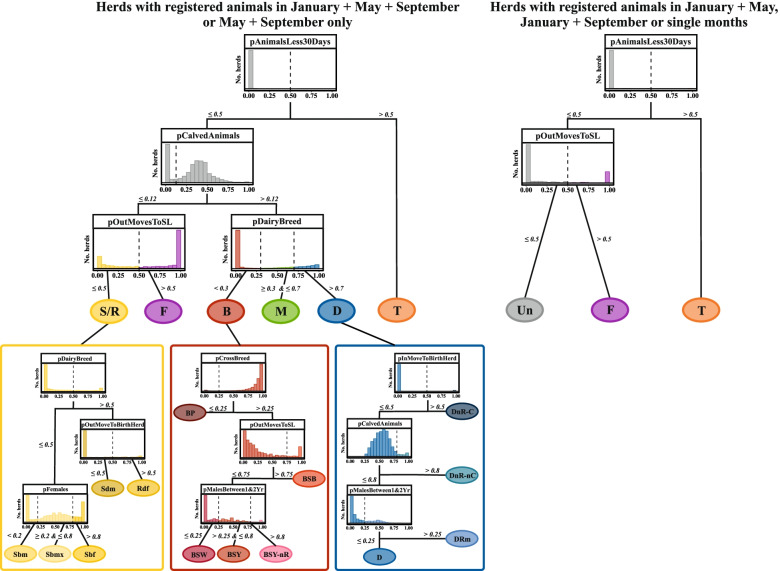
A decision tree for the classification of the Irish cattle sector adapted from [11]. Histograms represent herds remaining at the respective node (decision variable). Numbers and dashed line demarcate thresholds for class assignment. Main herd types: dairy (D), beef (B), mixed (M), store/rearing (S/R), fattening (F), and trading (T) herds. Dairy sub-types: dairy (D), dairy no rearing—contract (DnR-C), dairy no rearing—no contract (DnR-nC), dairy rearing male calves (DRm). Beef sub-types: beef pedigree (BP), beef suckling to weanlings (BSW), beef suckling to youngstock (BSY), beef suckling to youngstock—no rearing (BSY-nR), beef suckling to beef (BSB). Store/rearing sub-types: store dairy males (Sdm), store beef males (Sbm), Store beef females (Sbf), store beef mixed (Sbmx), rearing dairy females (Rdf). In each of the years between 2015 and 2019, an average of 95% of Irish herds were classified by the left tree, 5% by the right. A detailed description of the classification variables is provided in additional file 1

The next step was to assign each herd to the different herd management types identified in Ireland using a recently published classification concept presented in [11]. In their study [11] present a new approach for the classification of herd types in livestock systems by coupling expert knowledge and a machine-learning algorithm called self organising maps. The authors apply their approach to the cattle sector in Ireland and provide a data-driven classification tree using decisions derived from regular livestock registration data (AIM database). The resulting decision tree was used in this study to classify herds (see Fig. 1). The process of using self-organising maps to variables and decisions for class discrimination is provided in [11] but in the following a brief description is given.

Self-organising maps are an unsupervised learning algorithm used to project high-dimensional input data onto a low-dimensional map while preserving the topological properties of the input data [13]. Due to the nature of the algorithm data points that are similar in structure are mapped into nearby regions, allowing for a visual identification of clusters/classes in the output (a map). In terms of applying this algorithm to aggregated, multi-dimensional herd data, this means that herds that are similar in structure (demographic and transport characteristics) are clustered close to each other, allowing for visual identification of classes (herd types) with similar structural patterns. In the development of the classification tree, variables were added to the algorithm iteratively and through the use of expert knowledge, and their distributions were made visible across all herds whilst simultaneously accounting for the similarity of input records across all other variables. The iterative introduction of variables has led to the identification of clusters in the distributions of the data, indicating that the demographic structure of corresponding herds has been shaped by different livestock management practices.

The original decision tree after [11] to classify Irish cattle herds was based on data from those herds which either contained registered animals at all three time points each year or during both May and September (left tree in Fig. 1). In this study however, we extended the classification tree to also include herds that have animals registered at only one time point in January, May or September, or at only two time points (either January and May or January and September) hereafter non-continuous herds (right tree in Fig. 1).

In brief, the complete classification procedure was: First, all herds with registered animals in May and September or at all three time points were classified as before (left part of the tree). Second, the so-called non-continuous herds were classified. For these latter herds, most of our classification parameters cannot be calculated (see Additional file 1), and therefore these herds are now classified (right part of the tree) solely based on one demographic variable (proportion of purchased animals that leave the herd within 30 days) and one transport variable (proportion of animals that move to slaughter). Those herds that still could not be assigned to an enterprise type were termed unclassified (Un), representing an eighteenth herd sub-type.

The classification (sending each herd through the tree) was performed separately during each year of interest to account for possible changes in classification of the enterprise type between years.

## Results

### Main herd types and their distribution

**Fig. 2:**
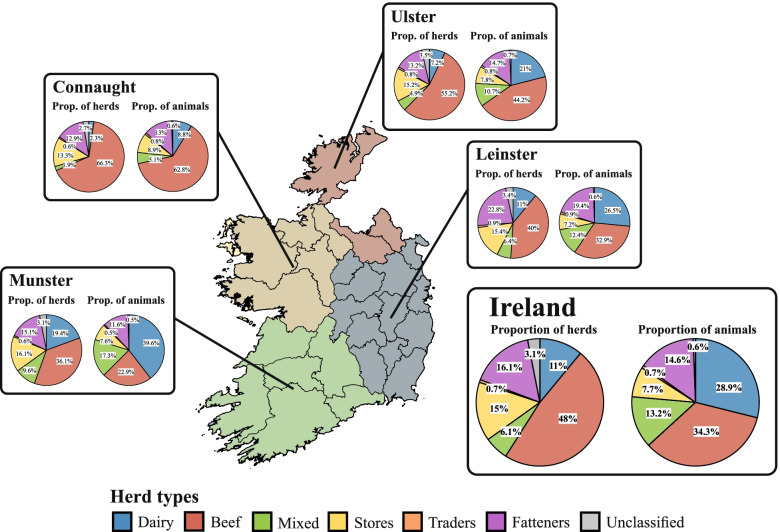
The distribution of cattle herd types, both in Ireland and in the four provinces (Connaught, Leinster, Munster, Ulster) in 2019. The left pie charts show the proportion of herds of each type in 2019, and the pie charts on the right the proportion of animals in herds of each type

**Fig. 3:**
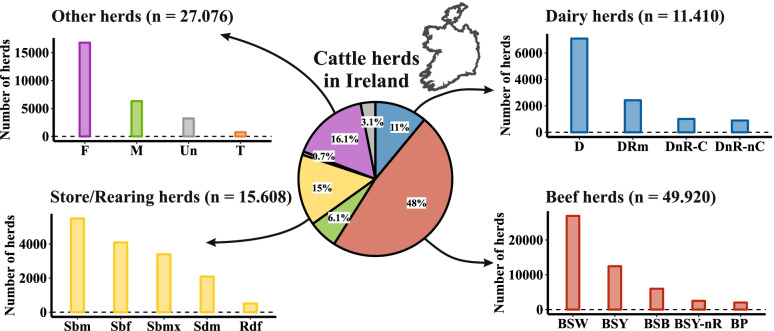
The distribution of cattle herd types and subtypes in Ireland during 2019. Abbreviations for the herd subtypes are as follows: D – dairy, DRm – dairy rearing male, DnR-C – dairy non rearing (contract), DnR-nC – dairy no rearing (non-contract), BSW – beef suckling to weanling, BSY – beef suckling to youngstock, BSB – beef suckling to beef, BSY-nR – beef suckling to youngstock no rearing, BP – Beef pedigree, Sbm – store beef males, Sbf – store beef females, Sbmx – store beef mixed, Sdm – store dairy males, Rdf – rearing dairy females, F – fattening, M – mixed, Un – unclassified, T – trading

The Irish cattle population is represented by seven main herd types, whose distributions at national and provincial levels are shown in Fig. 2 (left pie charts), and 18 subtypes (Fig. 3). Throughout Ireland, as well as in the four Irish provinces, beef herds are the most common herd type, accounting for 48% of all herds in 2019. The proportion of dairy herds is relatively low at 11%, but these herds are larger on average, which is reflected in the proportion of animals registered in these herds (28.9% nationally in 2019; Fig. 2, right pie charts). 6.1% of all herds in Ireland were classified as mixed herds, i.e., enterprises with both beef and milk production (with between 33 and 66% of the herd being of a dairy breed). When combined with those classified as dairy, a total of 17.631 herds were enterprises with an element of milk production. The store/rearing herd group accounted for 15% of the total herd population. These herds are non-breeding and specialise in purchasing young animals from beef and dairy herds. In store herds, these animals are collected and reared before being sold, primarily to fattening herds for finishing (see below). Fattening herds are the third most common herd category in Ireland (16.1%). These are finishing herds where cattle are primarily fed from grass. Trading herds (0.7%), as well as unclassified herds (3.1%), make up only a small proportion of the herds, both nationally and within individual provinces.

Structural differences in the composition of the cattle population, based on assigned herd type, were found between the provinces (Fig. 2). The distribution of herd types in Munster and Leinster is similar to the overall national situation, whereas the beef sector clearly dominates in Connaught and to a lesser extent in Ulster. Especially in Connaught, the dairy industry is underrepresented compared to the situation nationally.

### Herd subtypes

Figure 3 details the distribution of Irish herds in 2019 by the 18 herd subtypes to which they were assigned. In the following we describe the different herd subtypes (see also [11] for further details).

#### Dairy herds

Among herds classified as dairy herds (*n* = 11.000), four subtypes were identified, reflecting their differing management practices (Fig. 3). The most common dairy herd type (*n* = 6.000) sells their male calves within a few weeks after birth (subtype D), while sufficient female calves are kept and reared as replacement heifers. However, in 2019 more than 2.000 herds also reared their male calves and were classified as subtype dairy rearing male (DRm). Non-rearing dairy herds are also present in the Irish dairy sector, which sell most or all their calves and comprise two further dairy subtypes. Dairy non-rearing contract herds (DnR_C) comprised approximately 1.000 herds in 2019. These herds move their female calves to external contract rearing farms (Rearing dairy females; Rdf) where they are reared and later inseminated before returning to their birth herd as in-calf heifers.

Dairy non-rearing non-contract herds (DnR_nC) which source replacement cows through the purchase of non-homebred animals.

#### Beef breeding herds

Beef breeding herds (commonly referred to in Ireland as suckler herds) are by far the most common herd type in Ireland, with almost 50.000 herds registered (a herd with one or more animals) in 2019 being assigned to this class (Fig. 3). Five different subtypes of beef herds have been identified, which are consistent with recognised production systems which differ in their management characteristics including when animals are sold to further production. Beef suckling to weanling (BSW) producers maintain a herd of cows and raise calves from birth to weaning, with the majority sold as weanlings at autumn sales during September and October while a proportion of female calves is kept as heifer replacements. The beef suckling to youngstock (BSY) subtype is similar to BSW, including retaining a proportion of females as replacements, with the key difference being that calves are kept for a longer period, to allow weaned calves to gain weight prior sale. These animals are usually yearlings (12–20 months of age) by the time they leave their birth herd. Non-rearing suckling to youngstock (BSY-nR) herds are a variation of the BSY herd type, with the difference that female calves are sold after weaning and replacement bred females are purchased. The suckling to beef (BSB) herds follow the full beef production cycle, from birth through to the age of slaughter. Finally, representing only a small proportion of the beef sector in Ireland, beef pedigree (BP) herds are an important source of pedigree breeding stock to other commercial cattle producers in both the dairy and beef sectors.

#### Store/rearing herds

In 2019, more than 15.000 store or rearing herds were registered in the Republic of Ireland (Fig. 3). These herds are non-breeding, and focus on the rearing of young animals from the beef and dairy sectors. The five subtypes of store/rearing herds differ primarily regarding the source and sex of animals they purchase. Three of these subtypes purchase primarily from beef breeding herds and are further differentiated by their sex composition: store beef males (Sbm), store beef females (Sbf) and store beef mixed (Sbmx) systems (Fig. 2). These herds purchase beef animals as weanlings and rear them until they are sent to further production. Store dairy male (Sdm) herds typically purchase young male dairy and/or crossbred calves from dairy herds for rearing before being sold into further production. Finally, rearing dairy female (Rdf) herds introduce young female dairy calves, which are reared and bred before being returned, as bred heifers, to their birth herd i.e. DnR-C.

#### Other herds

Fattening (F) herds are confinement feeding operations where cattle are fed primarily finishing. More than 15.000 registered herds were classified as fattening in Ireland (16%) in 2019. Mixed (M) herds are dual purpose operators and produce milk, but also have another cattle enterprise, focused on beef production. Trading (T) herds are characterised primarily on the basis of having a very low proportion of animals that remain in them for more than 30 days. While they represent a very small proportion of all herds (0.7%), they play an important role in animal movements between other herd types and from other herd types to slaughter and export. Herds that could not be assigned to an enterprise type by our classification tree were termed unclassified. The majority of these are “seasonal” herds with registered animals at only single points of time in the year.

### Temporal trends in Irish cattle herd numbers

**Fig. 4:**
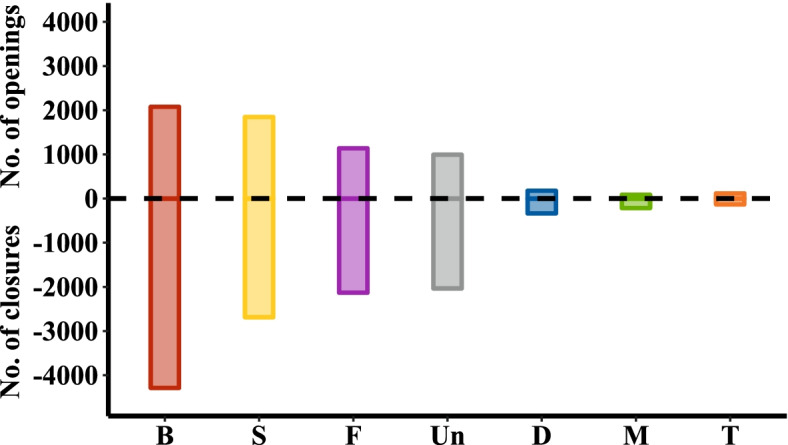
Total number of herd openings and closures between 2015 and 2019 by enterprise type. Abbreviations for the main herd types are as follows: B – beef, S – store, F – fattening, Un – unclassified, D – dairy, M – mixed, T – trading

The total number of eligible herds (with at least one animal registered) declined by 4.9% between the start and finish of the observation period, from 109.413 in 2015 to 104.014 in 2019. The total number of herds that ceased trading (closures) and were established (openings) between 2015 and 2019, by herd type, is presented in Fig. 4. A large proportion of herd closures (herds that had no registered animals compared to a previous year) were evident in beef herds. Of the more than 11.000 herds that ceased trading between 2015 and 2019, more than 4000 were beef type herds. During the same period, however, slightly more than 2000 new beef herds were established. There was also a relatively large number of closures in Store, Fattening and Unknown herds, but the majority of these closures were compensated for by new herd establishments. We allow remarking that it is not apparent from the herd data whether some of the herd closures are changes in herd numbers.

**Fig. 5:**
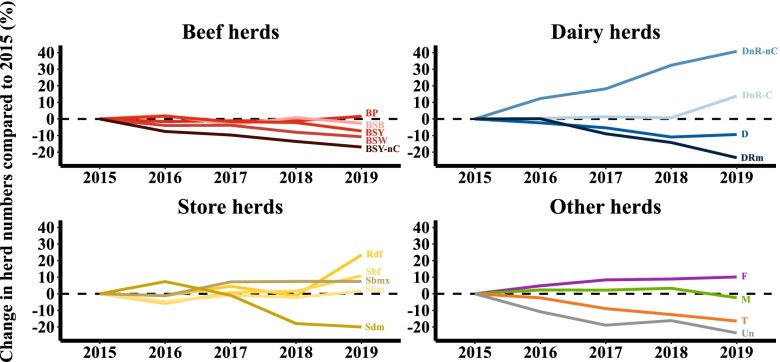
Proportional change in herd number per sub-herd type from 2015 to 2019. Herd type abbreviations as in Fig. 2

The data from Fig. 4 is shown again in Fig. 5, but with the percentage change in the number of herds assigned to each sub-herd type presented over the entire period of interest. Here again, the drop in the number of beef herds is evident, which is reflected in almost all subtypes, being greatest in BSY-nR herds. Compared to 2015, the number of dairy (D) and dairy rearing male (DRm) herds has also decreased. However, since 2015, a steady increase in the dairy non-rearing (DnR-nC & DnR-C) herds has been observed in the data, including a notable growth in DnR-C herds since 2018. The increase in DnR-C herds is also reflected in the growth in the number of rearing dairy female (Rdf) herds, where the female calves from the dairy non-rearing herds (DnR_C) are reared and inseminated. Between 2018 and 2019, the number of Rdf herds increased by almost 20%.

**Fig. 6:**
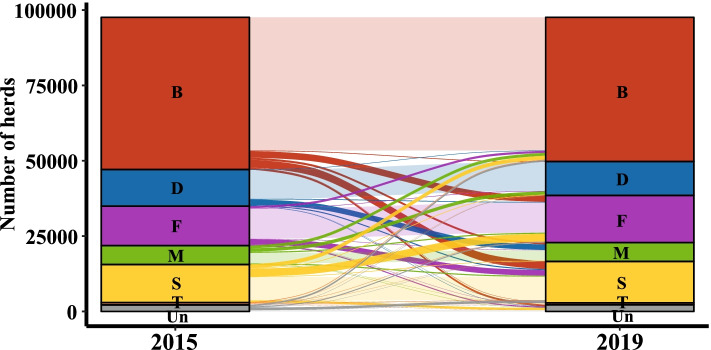
Total number of changes in main enterprise type between 2015 and 2019. The figure refers to herds with registered cattle in both years (2015 and 2019) and illustrates the number of herds per type that have changed their main enterprise type. Abbreviations for the main herd types are as follows: B – beef, S – store, F – fattening, Un – unclassified, D – dairy, M – mixed, T – trading

Figure 6 refers to herds with registered cattle in both years 2015 and 2019 and illustrates the number of herds per type that have changed their main production system. Of the 97.585 herds active (with at least one animal registered) in 2015 and 2019, 21.906 (22.4%) herds have changed their production type. However, no systematic changes in enterprise type can be identified from the data (i.e. a general move to one specific enterprise type). More frequent changes in enterprise type were observed from beef herds to fattening and store herds. Furthermore, a slight shift from dairy herds to mixed herds is visible in the data.

### Temporal trends in Irish cattle population numbers

**Fig. 7:**
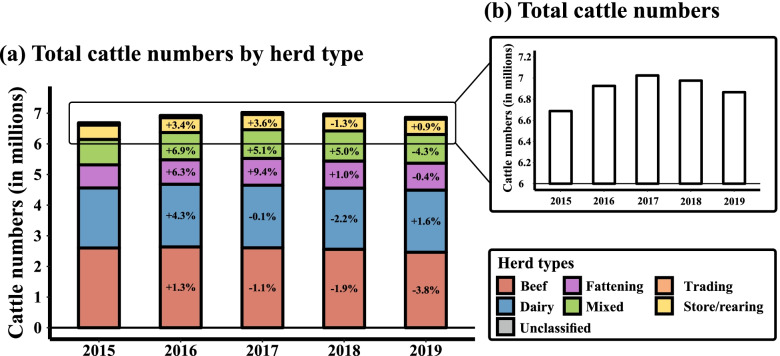
Total cattle numbers in Ireland between 2015 and 2019 per (**a**) herd type and (**b**) in total. The percentages in the bars show the average percentage change of the total number of animals compared to the previous year

Figure 7 presents trends of the Irish cattle population numbers by head from 2015 to 2019 (September data). Irish cattle numbers rose by about 5.2% (~ 340.000) between 2015 and 2017, peaked in 2017 at more than seven million before subsequently falling by more than 150.000 heads in 2019.

In beef herds, a steady decline in the number of animals has been observed since 2016. There were more than 2.64 million animals registered in beef herds in 2016, falling by 7.6% to 2.44 million in 2019.

A slight but positive increase in cattle numbers was observed in the Irish dairy sector, increasing by 3.5% from 1.95 million in 2015 to 2.02 million animals in 2019 (see also Additional file 3).

### Trade flows between herds and movements to slaughter

**Fig. 8:**
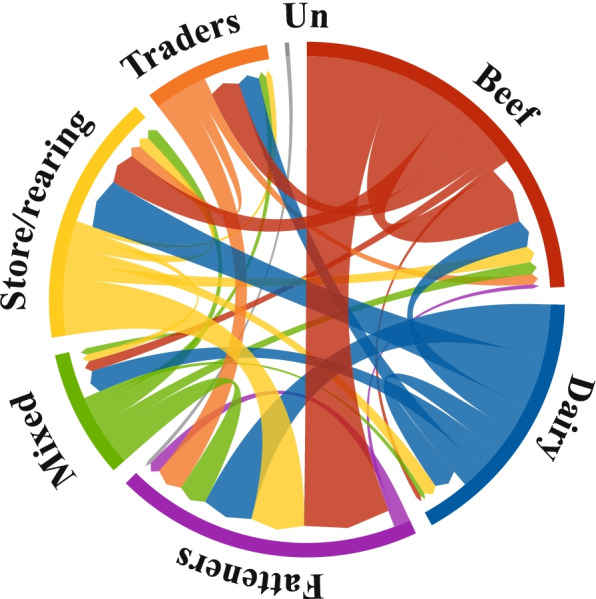
Between-herd movement flows between the main herd types in Ireland in 2019. The thickness of the arrow reflects the number of movements that occurred

There were more than 2.516.981 individual movement events relating to 2.051.184 animals between Irish cattle herds in 2019, excluding movements to slaughter and exports (Fig. 8). We have created centred alluvial plots to show the movement flows between herd types, using different colours to represent the movement network (Fig. 8). The thickness of the arrows is a measure of the movement volume. The circle segment of a herd type is separated into the in and out moves proportionally to the respective volume.

Animal movements took place between all herd types in 2019. More animals were sold than bought from the breeding beef, mixed and dairy herds. From these herds, animals were mainly moved to store or fattening herds. In the store and trading herds, the ratio between in- and outflows is balanced. In contrast, while cattle move into fattening herds from all of the other herd types, very few out-moves were recorded because the final move to slaughter is not included in this analysis. With regard to the out-moves from fattening herds, it is noticeable that they occur more frequently between fattening herds, which suggests a two-stage fattening system in some herds. Although trading herds represent a very small proportion of herds in Ireland (0.7%), they are relatively well represented in the trading network, with more than 200.000 in- and out-moves, respectively (excluding movements to slaughter and export via trading herds).

**Fig. 9:**
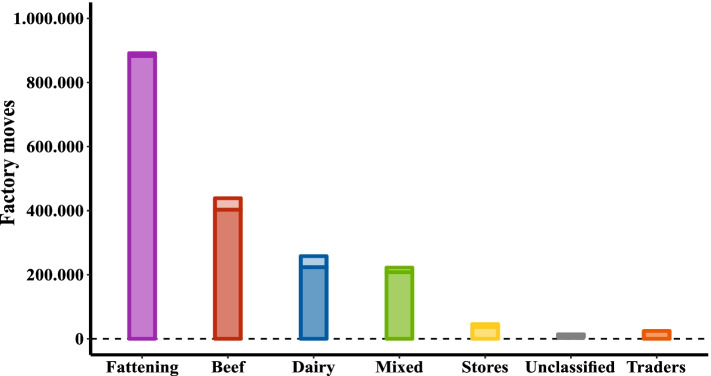
Total number of movements directly to slaughter (factory moves) by herd type in 2019. Dark shaded bars show direct movement while lighter shaded bars show factory moves via trading herds

In 2019, a total of 1.895.279 cattle were finished (that is, moved to slaughter; Fig. 9). This included movement of animals directly from fattening herds (*n* = 891.386 movements), from beef herds (438.846), from dairy herds (258.038), and from mixed herds (222.224). Only a small proportion of animals destined for slaughter were transported via trading herds to the slaughterhouse (lighter shader portions in Fig. 9).

## Discussion

The cattle sector is the most important economic production unit of the Irish farming and agri-food sector [1]. Providing a structured overview of Irish cattle herds by enterprise type, including their populations and interactions, is a valuable information basis for decision-makers. Despite the comprehensive data availability through a national cattle database (AIM database), there has not previously been a comprehensive overview of the Irish cattle population structured by farm type. To the author’s knowledge, this is the first study to provide a detailed visual overview of the entire Irish cattle sector.

Substantial differences in the management of different herd production types has long been recognised (e.g. [4] for the beef sector), but could not be quantitatively described. In the absence of uniform classification standards, it has not been possible – prior to the development of the current methods – to assign herd type or subtype in a consistent, transparent and data-driven manner. With the work presented in [11] this problem has now been overcome and we are now able to present quantitative information structured by herd enterprise type that e.g. allows us to observe changes in the Irish cattle population structured by herd type.

The results of this study have shown that the overall number of active breeding herds in Ireland has slightly decreased between 2015 and 2019. However, while the number of herds has decreased, the total number of animals in breeding herds has increased. In particular, the data shows an increase in the number of bovines in dairy and mixed herds (by more than 180.000 animals). This increase was facilitated by the abolition of milk quotas in 2015. Since then, milk production has increased significantly [2], which has led to constraints in the availability of land around milking parlours, farm facilities and labour. These constraints have apparently resulted in an increased expansion of alternative systems, leading, as one example, to an increase in contract rearing of dairy heifers (see Fig. 5). Contract heifer rearing involves the movement of the replacement heifers from the owner’s farm to another farm for rearing under a contract agreement [5]. The replacements may be moved from, and returned to, the owner’s farm at different ages depending on the individual agreements made. With the heifers now contract reared on another farm, there is only one group of animals (dairy cows) to be managed on the dairy farm, maximizing the milk output generated from this block of land. According to [14] this allows for increased efficiency and ease of management. With the classification at hand, for the first time it is possible to identify the extent to which contract rearing is being used, and to follow its increasing practice. In 2019, more than 1000 herds were non-rearing contract herds (DnR-C), i.e. herds that send their female calves to external contract rearing farms (Rearing dairy females; Rdf).

The pattern of activity within the Irish cattle system that is described in section “Herd subtypes” is also evident from the analysis of between-herd movements and movements to slaughter (see Fig. 7). For example, there are major movement flows from dairy to store herds (male calves and, to a lesser extent, female calves for rearing, both with a significant number of cross bred animals). The classified herd data also highlights the role of fattening herds in the Irish production system. These herds are gathering points for animals from herds of all production types and are therefore good potential candidates as points of surveillance regarding the health status of much of the Irish cattle population. It is also evident from the data that trading herds (T) play a significant role in the Irish cattle movement network. Even though trading herds represent only 0.7% of Irish herds, a significant proportion of animals pass through these herds, with moves in primarily from beef herds followed by dairy herds, and moves out to fattening herds, with smaller numbers to store/rearing and beef herds (see Fig. 8). Compared to the other herds, trading herds have substantially more trading partners (see Additional file 2), which further highlights their disproportionate role in movement of animals and potentially disease. Overall, the classified movement data clearly shows that all herd types in Ireland are in contact with each other through animal movements. We refer to [8, 9] for more details on Irish cattle movements.

From a methodological point of view, the allocation of herds to a certain production type is a simple and transparent process, as straightforward classification rules are followed. Further, the decision tree on which this work is based (see also [11]) can be applied at all spatial and temporal scales, offering a variety of benefits for a wide range of applications, some of which are briefly described below.

First, and possibly most relevant, there are benefits from an epidemiological perspective for strategic planning of control and surveillance measures. In the context of the new European Animal Health Law (AHL), the herd management type has become an important criterion for consideration when shaping the sampling strategy of national surveillance and control programmes [15]. This is particularly relevant to a current application in Ireland, namely the detailed design, including conceptual evaluation, of a national programme to eradicate bovine herpesvirus type 1 (BoHV-1) [16]. The AHL prescribes certain rules on how herds can acquire and maintain an infection-free status. These rules are associated with a variety of testing regimes, the applicability (and cost) of which in turn depend on herd management. As one example, the use of bulk tank milk testing, either alone or supported by blood sampling, is only permitted in herds that meet certain thresholds regarding the proportion of lactating cows, females intended for milk production and of male animals. A typical self-breeding Irish dairy herd (D) would not meet this criterion, whereas this would be possible in non-rearing dairy herds (DnR-C and DnR-nC). Through the quantification of these management details, it is now possible to estimate in a targeted way how many tests are needed per test type (e.g. tests on blood or individual milk samples compared with or bulk tank milk samples) and year. This allows a more accurate cost estimate of intervention measures and better forecasting and development of laboratory capacity.

In this study, we have also shown how the classification can be used to analyse transport data in a structured way according to enterprise type, providing a robust framework for a generalised overview of inter-herd trade dynamics. There has been considerable work to date on representing Irish cattle movements (e.g. [9]) and analysing their spatial and network characteristics (e.g. [8]). Incorporating herd type in this kind of analysis will be particularly helpful in identifying the recurrent transport routes that are potentially most relevant for the spread of infectious diseases. These more detailed insights will make it easier to understand infectious disease spread in the national cattle population, to inform animal health policy and to implement efforts for improved management both of endemic and epidemic diseases. This increased granularity in terms of herd types and subtypes will also enhance risk factor studies.

In addition, the overview and associated classification presented in this study will form the basis for a number of future comparative studies. With the classification system at hand it is now possible, for example to compare the emission of greenhouse gases for different farming systems between and within main herd types. This was not possible before, as to the authors knowledge no classification system exists that was classifying production types beyond dairy beef and mixed. Formally, with the new classification system it is possible to quantitatively compare e.g. Dairy non-rearing contract herds (DnR) against typical self-breeding dairy herds (D) in relation to economic output, greenhouse gas emission and biomass distribution, making it possible to identify specialized farming systems that might be more efficient.

## Supplementary Information

**Additional file 1.** Variables for the classification of herd types.

**Additional file 2.** Number of trading partners per enterprise type.

**Additional file 3.** Irish cattle herd characteristics by enterprise type.

## Data Availability

The dataset used in this study is the private property of farmers located in the Republic of Ireland. The data was made available for research purposes and cannot be made publicity available.
