# Supplementary material for: The Irish cattle population structured by enterprise type: overview, trade & trends
Source: Ir Vet J. 2022 Apr 4;75:6. doi: 10.1186/s13620-022-00212-x (PMC8978404; doi:10.1186/s13620-022-00212-x)
Supplement: Supplementary file 1 — Additional file 1. Variables for the classification of herd types. [file 13620_2022_212_MOESM1_ESM.docx]

Additional file 1: Variables for the classification of herd types

| Group | Variable | Description | Calculation | Rationale |
| --- | --- | --- | --- | --- |
| Demographic variables | **pFemaleAnimals** | Proportion of females in a herd in May, relative to herd size | $\frac{N females May}{herd size May}$ | To get an overview of the sex distribution |
|  | **pDairyBreed** | Proportion of dairy breeds in a herd in May, relative to herd size | $\frac{N dairy breed May}{herd size May}$ | To get an overview of the breed distribution |
|  | **pCrossBreed** | Proportion of dairy-beef cross breeds in a herd in May, relative to herd size | $\frac{N cross breed May}{herd size May}$ | To extract pure breed herds |
|  | **pCalvedAnimals** | Proportion of animals in a herd that have ever calved by September, relative to herd size | $\frac{N calved animals September}{herd size September}$ | To differentiate breeding and non-breeding herds |
|  | **pMalesBetween1&2Years** | Proportion of males between one and two years of age in a herd in May, relative to all animals between one and two years of age | $\frac{N males between 1 and 2 May}{N animals between 1 and 2 May}$ | To extract herds that keep their males for fattening |
|  | **pAnimalsLess30Days** | Proportion of purchased animals in a herd that remain for less than 30 days before being sold, relative to all out moves in a year | $\frac{N animals less 30 days}{N out moves}$ | To extract dealer/trading herds |
| Transport variables | **pOutMovesToSL** | Proportion of animals in a herd sold to the slaughterhouse per year, relative to maximum herd size (maximum herd size = largest herd size during one of the three sampling dates) | $\frac{N out moves to SL}{maximum herd size}$ | To differentiate between herds that sell their animals directly to the slaughter and those that use dealer herds etc. |
|  | **pOutMovesToBirthHerd** | Proportion of animals in a herd moved out which are returning to their birth herd, relative to all out moves (calculated over one year) | $\frac{N out moves to birth herd}{N out moves}$ | To extract contract rearing herds |
|  | **pInMovesToBirthHerd** | Proportion of animals in a herd moved in which are returning to their birth herd, relative to all in moves (calculated over one year) | $\frac{N in moves to birth herd}{N in moves}$ | To extract non-rearing herds that use a contract rearer |
