# Supplementary material for: The Irish cattle population structured by enterprise type: overview, trade & trends
Source: Ir Vet J. 2022 Apr 4;75:6. doi: 10.1186/s13620-022-00212-x (PMC8978404; doi:10.1186/s13620-022-00212-x)
Supplement: Supplementary file 2 — Additional file 2. Number of trading partners per enterprise type. [file 13620_2022_212_MOESM2_ESM.docx]

Additional file 2: Number of trading partners per enterprise type


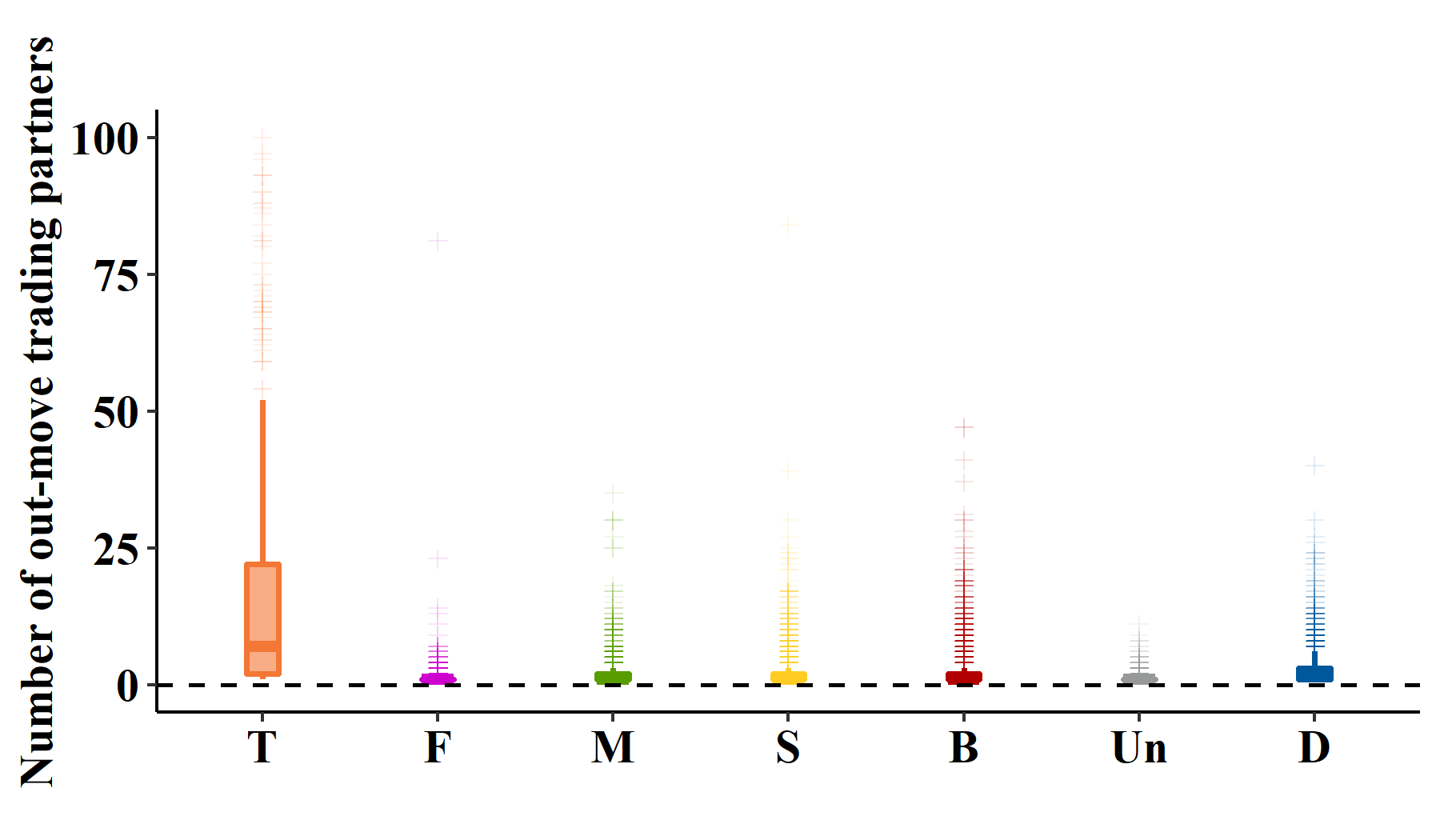

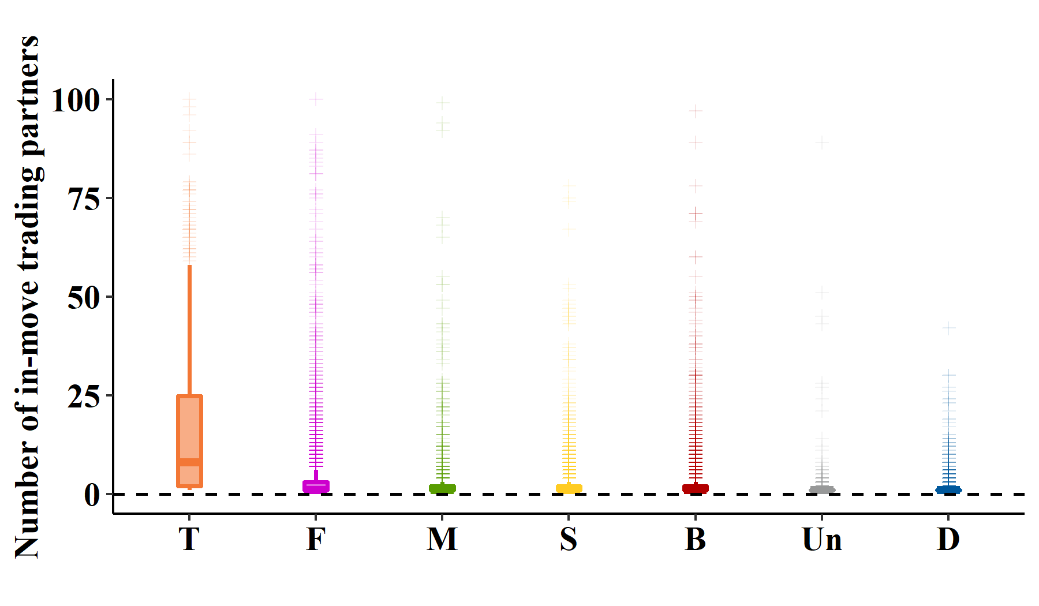


Total number of out- and in-move trading partners per enterprise type in 2019. Abbreviations for the main herd types are as follows: T – trading, F – fattening, M – mixed, S – store, B – beef, Un – unclassified, D – dairy.

**The distribution of the number of trading partners associated with in-and out-moves for each herd over the course of an entire year (2019). On average, trading herds have the most trading partners in both in- and out-moves.**
