# Supplementary material for: The Irish cattle population structured by enterprise type: overview, trade & trends
Source: Ir Vet J. 2022 Apr 4;75:6. doi: 10.1186/s13620-022-00212-x (PMC8978404; doi:10.1186/s13620-022-00212-x)
Supplement: Supplementary file 3 — Additional file 3. Irish cattle herd characteristics by enterprise type. [file 13620_2022_212_MOESM3_ESM.docx]

Additional file 3: Irish cattle herd characteristics by enterprise type

Abbreviations for the herd subtypes are as follows: D – dairy, DRm – dairy rearing male, DnR-C – dairy non rearing (contract), DnR-nC – dairy no rearing (non-contract), BSW – beef suckling to weanling, BSY – beef suckling to youngstock, BSB – beef suckling to beef, BSY-nR – beef suckling to youngstock no rearing, BP – Beef pedigree, Sbm – store beef males, Sbf – store beef females, Sbmx – store beef mixed, Sdm – store dairy males, Rdf – rearing dairy females, F – fattening, M – mixed, Un – unclassified, T – trading.

|  | Enterprise type | Number of herds in 2019 | Prop. change in herd numbers since 2015 | Total animals in 2019 | Prop. change in total animals since 2015 | Avg. herd size in 2019 |
| --- | --- | --- | --- | --- | --- | --- |
| Dairy | D | 7.086 | -9.3% | 1.325.632 | 6.7% | 187 |
|  | DRm | 2.424 | -23.3% | 569.931 | -11.2% | 235 |
|  | DnR-C | 1.009 | 13.7% | 269.905 | 27.1% | 267 |
|  | DnR-nC | 891 | 40.7% | 77.645 | 70.1% | 87 |
| Beef | BSW | 26.947 | -10.7% | 1.072.478 | -7.4% | 40 |
|  | BSY | 12.454 | -7.2% | 847.319 | -6.9% | 68 |
|  | BSY-nR | 2.497 | -16.9% | 94.996 | -19.9% | 38 |
|  | BSB | 5.996 | -2.5% | 549.582 | 5.6% | 91 |
|  | BP | 2.026 | 1.6% | 95.481 | 13.1% | 47 |
| Store/rearing | Sbm | 5.501 | 1.6% | 142.463 | -1.1% | 26 |
|  | Sbf | 4.098 | 10.8% | 101.184 | 10.7% | 24 |
|  | Sbmx | 3.399 | 7.4% | 176.019 | 18.8% | 52 |
|  | Sdm | 2.094 | -20.1% | 137.546 | -5.0% | 66 |
|  | Rdf | 516 | 23.4% | 42.504 | 49.1% | 82 |
| Other | M | 6.321 | -2.3% | 1.027.117 | 14.1% | 162 |
|  | F | 16.786 | 10.1% | 1.135.491 | 20.3% | 68 |
|  | T | 748 | -16.4% | 56.869 | 5.5% | 76 |
|  | Un | 3.221 | -23.6% | 43.525 | -33.3% | 13 |
